# Implications of food and nutrition security on household food expenditure: the case of Malaysia

**DOI:** 10.1186/s40066-022-00367-4

**Published:** 2022-04-07

**Authors:** Kerry Kh’ng, Ching-Cheng Chang, Shih-Hsun Hsu

**Affiliations:** 1 Department of Agricultural Economics, National Taiwan University, Taipei, Taiwan; 2 Institute of Economics, Academia Sinica, Taipei, Taiwan

**Keywords:** Food and nutrition security, Food cost, Nutrient intake, Malaysian Adult Nutrition Survey, Goal Programming

## Abstract

**Introduction:**

Food security is attracting more attention in Malaysia not only at the national level that concern toward the enhancement of food self-sufficiency but also at the individual level which concerns more on nutrition and health. The economic recession triggered by the COVID-19 pandemic has brought the food and nutrition security challenge to the higher priority. In this study, we assessed the feasibility of encouraging a healthy eating plan by taking into account two important elements, food cost and nutrient intake, to help tackle the food and nutrition insecurity challenges at the individual level.

**Method and materials:**

This study used a goal programming model with dietary intake data from Malaysian Adult Nutrition Survey reports to develop food plans that can improve nutrition quality without increasing food cost. Missing data, such as nutrient compositions and food prices, were collected separately from existing governmental and non-governmental sources. Benchmark nutrient intakes were derived from Malaysian Dietary Guidelines and Malaysian Recommended Nutrient Intakes reports, whereas benchmark costs were estimated by mapping food prices to dietary intake. The cost of healthier diets was also assessed to examine the acceptability of dietary changes for the low-income population.

**Results:**

The results showed that healthier diets following national dietary guidelines are achievable with reasonable food choices shift without changing the cost of meal plan. Greater intake of milk and vegetables (for more calcium) and smaller intake of seafood and egg products (for less protein) will contribute to raise diet quality and achieve more adequate nutrition. However, the cost attached to healthier food plan is still likely to be burdensome for the food-insecure and low-income population.

**Conclusions:**

Our results suggest that policymakers should implement income-relevant laws to cut poverty and improve the population’s dietary intake. Income growth as a result of better skills and education is needed to ensure that the real incomes of Malaysian are well sustained, and increased to help low-income population make better and healthier food choices.

## Introduction

The definition of food security was first introduced in the 1970s from the perspective of food supply to ensure that all people everywhere have sufficient food to eat [1]. This definition was expanded in 1996 to incorporate nutrition and cultural dimensions [2]. Unlike food security, nutrition security was interpreted from the viewpoint of food demand as “a person is considered nutrition secure when she or he has a nutritionally adequate diet and the food consumed is biologically utilized such that adequate performance is maintained in growth, resisting or recovering from disease, pregnancy, lactation and physical work” [3]. With the widespread recognition of the importance to include nutritional aspects into food security by international organizations such as FAO, UNICEF, and IFPRI, analysis has also expanded to consider both global and individual levels, as well as multiple forms of malnutrition [4]. Following the 2007–08 food price spikes, growing attention to the food self-sufficiency and greater awareness of the public health implications of malnutrition has in turn influenced food security policy in important ways [5].

For decades, food insecurity has always been correlated with poverty and health issues [6–9]. Although the incidence of poverty in Malaysia had significantly reduced [10, 11], it does not mean that Malaysian households are free from food insecurity situation. Many demographic indicators, such as sex, age, source of income, household type, homeownership, marital status, immigrant status, and aboriginal status, can be relevant to household food insecurity. Among them, household income was found to be a factor that driving significant impact to food insecurity [12]. Researchers also found that 13.4% of Malaysian adults tend to reduce the size of meals and skip main meals due to financial constraints [13]. Intervention measures, such as the National Plan of Action for Nutrition (NPAN), has been launched by the Malaysian government to ensure food security and nutrition security for all households and to prevent diet-related non-communicable diseases [14]. Strategies and activities have also been identified for implementation to ensure the availability of quality and safe food to all households at affordable prices. However, meeting healthy diet recommendations may involve substantial adjustment in dietary pattern which will also influence food purchase cost. In fact, about 17.3% of Malaysian household income are devoted to food consumption expenditures [15]. It accounts as the second highest percentage to overall basic necessities’ expenditure but as the highest for families in the rural areas. The high percentage of income committed to food consumption may cause low-income families especially vulnerable to food insecurity, poor nutrition, and leading to various health issues.

In March 2019, the Employees Provident Fund (EPF) of Malaysia released an expenditure guide for Malaysian individuals and families. The guideline indicated that an individual who is single and a public transport user required a budget of RM 1870 per month for his or her living [16]. The suggested budget on food was RM 550, equivalent to 29% of the overall budget and 50% of the minimum wage. The imbalance ratio of food budget to the minimum wage has stimulated our curiosity, for which we find that it is necessary to provide a better understanding of how healthy eating habits will impact the low-income group especially those who had been attached or is still attaching to the minimum wage. In addition, the recent COVID-19 outbreak has also brought the income issue to a higher level of concern, unemployment from either job losses or reduction in working hours has significantly affected the livelihoods of Malaysians [17], and hence consolidated our motive of investigation.

This study aims to evaluate the feasibility of encouraging a healthy eating habit that follows national recommended dietary guidelines to the low-income group in Malaysia through modeling the realistic diets by using a food-based goal programming optimization model approach. It is an approach extended from linear programming and was popularly adopted to develop economically feasible food plans while promoting healthy dietary patterns simultaneously [18–20]. It was also empirically proven as an efficient and effective method to solve dietary problems [21]. To the best of our knowledge, such application can be barely found in the case studies of Malaysia and researches that gauge the balance in between food and nutrition security at the individual or household levels are also limited in Malaysia. Hence, this study serves as a contribution to identify the diet plans at individual level by taking into considerations of the important linkage of economic accessibility, food availability, and food utilization to help realize local government initiatives as well as global initiatives for tackling food insecurity challenge for the low-income population.

## Method and materials

### Goal programming model

This study adopted the goal programming approach using dietary intake data from Malaysian Adult Nutrition Survey (MANS) of the year 2003 and 2014 to design food-based dietary recommendations for Malaysia. Goal programming is a tool for solving multiple-goal problems with an objective function to minimize the sum of absolute values of deviations from various goals [22]. The approach was applied [21, 23, 24] in formulating the optimal food plan which aims to improve nutritional intakes via more prudent food group and subgroups choices under cultural, habitual dietary patterns, and economic cost considerations.

The general structure of the model is as follows:

1 $${\text{Minimize }}Y = \sum\nolimits_{k = 1}^{m} {\left| {\left( {X_{k}^{{{\text{opt}}}} - X_{k}^{{{\text{obs}}}} /X_{k}^{{{\text{obs}}}} } \right)} \right|} , \quad{k = 1, \ldots , m},$$

subject to

2 $$N_{i}^{{{\text{low}}}} \le \sum\nolimits_{k = 1}^{k = m} {a_{ik} X_{k}^{{{\text{opt}}}} \le N_{i}^{{{\text{up}}}} , }\quad{ i = 1, \ldots , n},$$

3 $$\sum\nolimits_{k} {\left( {\begin{array}{*{20}c} j \\ k \\ \end{array} } \right)0.5 X_{k}^{{{\text{obs}}}} \le X_{k}^{{{\text{opt}}}} \le \sum\nolimits_{k} {\left( {\begin{array}{*{20}c} j \\ k \\ \end{array} } \right)0.95 X_{k}^{{{\text{obs}}}} ,\quad{ j = 1, \ldots , 7}} } ,$$

4 $$\sum\nolimits_{k = 1}^{m} {c_{k}^{{{\text{low}}}} X_{k}^{{{\text{obs}}}} \le \sum\nolimits_{k = 1}^{m} {\overline{c}_{k} X_{k}^{{{\text{opt}}}} \le \sum\nolimits_{k = 1}^{m} {c_{k}^{{{\text{up}}}} X_{k}^{{{\text{obs}}}} } } } ,$$

where subscript *k* denotes *m* food subgroups, *i* the *n* nutrients, and *j* the seven major food groups;

$Y$ denotes the objective variable to be minimized;

$X_{k}^{{{\text{opt}}}} :$ denotes the optimal daily intake quantity of food subgroup *k*;

$X_{k}^{{{\text{obs}}}}$: denotes the observed daily intake quantity of subgroup *k*;

$a_{ik } :$ denotes the amount of nutrient *i* in unit of each subgroup *k*;

$N_{i}^{{{\text{up}}}} , N_{i}^{{{\text{low}}}}$ denote the upper, lower amount of nutrient *i* required; and.

$\overline{c}_{k} ,c_{k}^{{{\text{up}}}} , c_{k}^{{{\text{low}}}}$ denotes the averaged, upper, lower price of food subgroup *k.*

The objective function aims to minimize the gap between the quantity of optimal food intake and the observed quantity of intake by the study population. To standardize the gap across different food groups, it is divided by the observed quantity consumed.

The objective function can be transformed into a linear function [21] with two sets of non-negative decision variables representing, respectively, the positive deviation $(D_{k}^{ + } )$ and negative deviation $(D_{k}^{ - } )$ from the observed food intake, as follows:

5 $${\text{If }}X_{k}^{{{\text{opt}}}} < X_{k}^{{{\text{obs}}}} {\text{, then }}D_{k}^{ - } = \left( {X_{k}^{{{\text{obs}}}} - X_{k}^{{{\text{opt}}}} } \right)/X_{k}^{{{\text{obs}}}} {\text{ and }}D_{k}^{ + } = 0,$$

6 $${\text{If }}X_{k}^{{{\text{opt}}}} > X_{k}^{{{\text{obs}}}} ,{\text{then }}D_{k}^{ - } = 0\,{\text{and }}D_{k}^{ + } = \left( {X_{k}^{opt} - X_{k}^{obs} } \right)/X_{k}^{obs} { }{\text{.}}$$

The linearized objective function (Y*) is then defined as follows:

7 $${\text{Minimize }}Y^{*} = \sum\nolimits_{k = 1}^{k = n} {\left( {D_{k}^{ + } + D_{k}^{ - } } \right) , k = 1,{ } \ldots ,{ }n{\text{, and}}}$$

8 $$D_{k}^{ + } , D_{k}^{ - } \ge 0.$$

Three sets of constraints are included. First, Eq. (2) ensures that the daily total nutrient intake meets the desired level for Malaysian adult population. Second, Eq. (3) ensures that the serving amount of each major food group is within the range from the 5th percentile (as a lower limit) to the 95th percentile (as an upper limit) of daily consumption. Third, Eq. (4) ensures that daily per capita food cost is within the boundary of observed levels to prevent food plan from incurring unreasonable cost.

In essence, the model is seeking an intervention that can encourage people to make better food plans with least possible changes in dietary habit and cost. The finding of the optimal food plan may not satisfy all the nutrient requirements. Nevertheless, more realistic, affordable, and healthier recommendations are made to the target population for nutrition promotion purpose.

### Food and nutrient intake data

The complete list of data sources for this study is provided at Appendix 1. The main data source on food intake was extracted from the published reports of two Malaysian Adult Nutrition Surveys in 2003 (MANS 2003) [25] and in 2014 (MANS 2014) [26]. MANS 2003 was a nationwide survey with a total of 6886 households in Peninsular Malaysia, Sabah, and Sarawak. It contains a total of 126 food items. MANS 2014 was the second survey with a total of 2973 households and 165 food items. Both surveys conducted with a stratified random sampling method with proportional allocation. Specifically, data retrieved were the estimated mean per capita food intake (in gram) by food items per day of the surveyed households.

Several steps were taken to convert the food intake data into nutrient forms before they are incorporated into the goal programming model. First, the mean food intake of 126 and 165 food items in MANS 2003 and MANS 2014 were, respectively, grouped into 77 and 76 subgroups at per capita basis following Malaysian Dietary Guidelines (MyDG) [27]. Second, the nutrient compositions of the mean food intake by 77 and 76 subgroups were estimated using Malaysian Food Composition Database (MyFCD) [28]. Other sources, such as Food and Nutrient Database from Taiwan [29], Nutritionix website, and many more, were also used for nutrient estimations for subgroups that were not available in MyFCD. Third, because the food intake constraints in the model were specified in serving units by 7 major food groups, the food intake data were further converted into food groups based on one single nutrient indicator of each food group [30]. The mapping of 77 and 76 subgroups with 7 food groups is shown in Appendix 2.

**Fig. 1:**
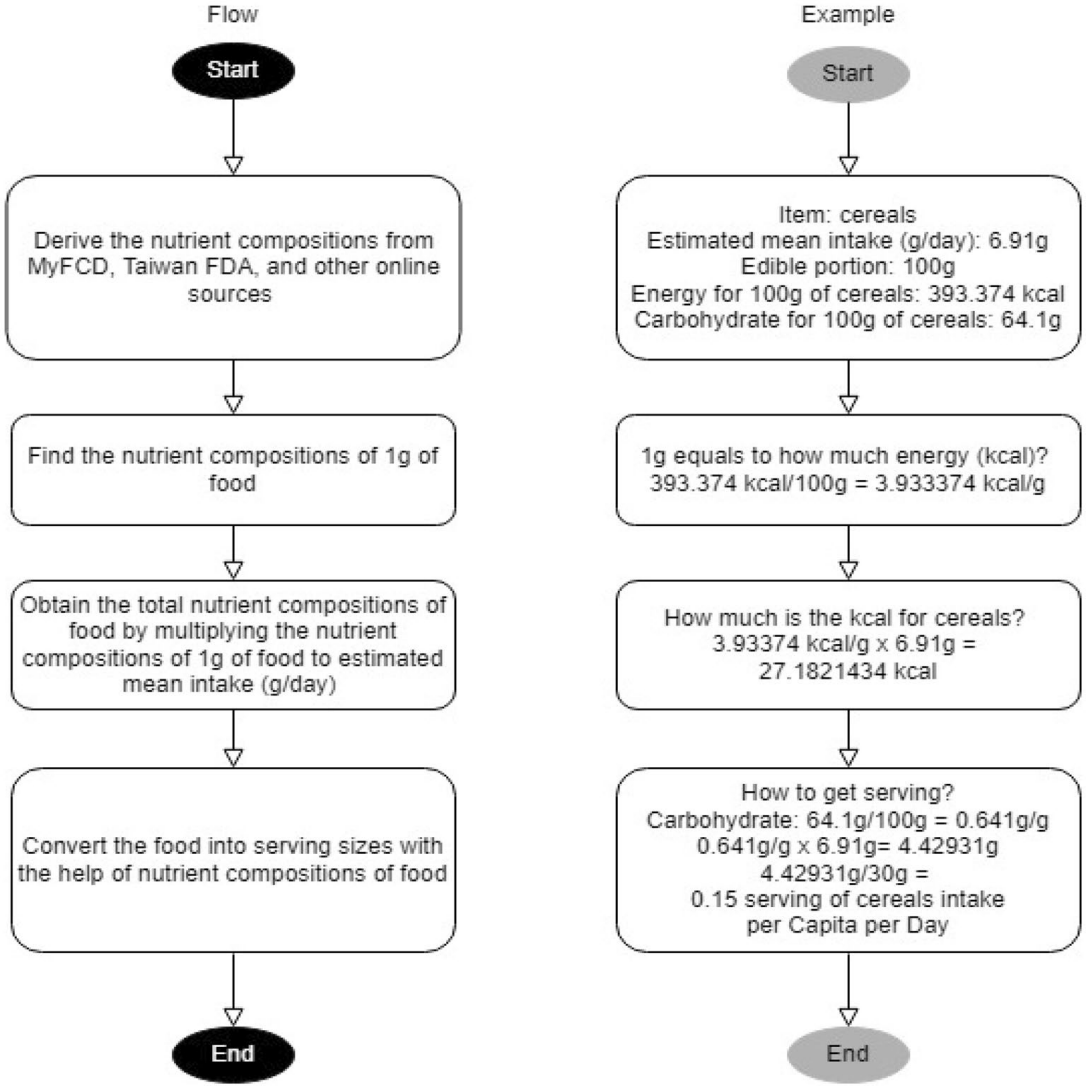
Flowchart of nutrient composition and serving size conversions

For instance, recommended intake for cereals and cereal products was based on 30 g of carbohydrates per serving per person. Hence, the mean servings for *bijirin* (cereals) subgroup are equal to its intake volume in gram divided by 30 g of carbohydrates and by summing up all the relevant food subgroup servings to form the food group serving. The overall conversion process is illustrated in Fig. 1.

### Upper and lower limit of food intake by food groups

The constraints of the goal programming model require the lower and upper limit of food intake in serving units and nutrient forms. For the serving units, data from Malaysian Dietary Guidelines (MyDG) of seven major food groups were used as the limits and they are listed in Table 1. Note that the lower limit is based on 2000 kcal per day instead of the original 1500 kcal available in MyDG taking into account the standard benchmark of 2100 kcal per day for adults as suggested by the United States Department of Agriculture [31].

**Table 1:** The lower and upper limit of daily per capita food intake in serving unit

Food groups	Lower limit	Upper limit
	2000 kcal	2500 kcal
A. Cereals and cereal products	6	8
B. Meat and meat products	1	2
C. Fish/seafood/eggs	1	1
D. Legumes and products	1	1
E. Milk and milk products	2	3
F. Vegetables	3	3
G. Fruits	2	2

Source: MyDG (2010)

Food group A was based on 30 g of carbohydrates per serving; B and C were based on 14 g of protein per serving; D and E were based on 7 g of protein per serving; F was based on 80 g per serving size [32]; G was based on 15 g of carbohydrates per serving

### Upper and lower limit of nutrient intake

For the constraints in nutrient forms, the upper and lower limits are listed in Table 2. They are averaged from the units derived from 2017 Recommended Nutrient Intakes (RNI) report of Malaysia [33]. Note that iron nutrient is excluded due to the difficulty in conversion to a single representation of total population.

**Table 2:** The lower and upper limit of daily per capita nutrient intake

Nutrient	Unit	Adult (Age: 20–59)	
		Lower limit	Upper limit
Energy	kcal	2100	–
Protein	% of energy	10	20
Fat	% of energy	25	30
Carbohydrate	% of energy	50	65
Calcium	mg	1200	2000
Sodium	mg	1500	2300
Vitamin A	μgRE	600	3000
Vitamin C	mg	70	2000
Vitamin B1	mg	1.2	–

Source: RNI [33]; Meade and Thome [31]

The lower bound is adequate intake, while “–” denotes that no upper bound is defined

### Food cost data

The food intake data of MANS did not include cost data. Therefore, the food cost data by subgroups are collected separately from various public and private online sources over three-year period of 2016, 2018, and 2019. For consistency purpose, the cost data are traced back using the Consumer Price Index (CPI) of Malaysia with the latest available figures derived from the Department of Statistics Malaysia (DOSM).

**Fig. 2:**
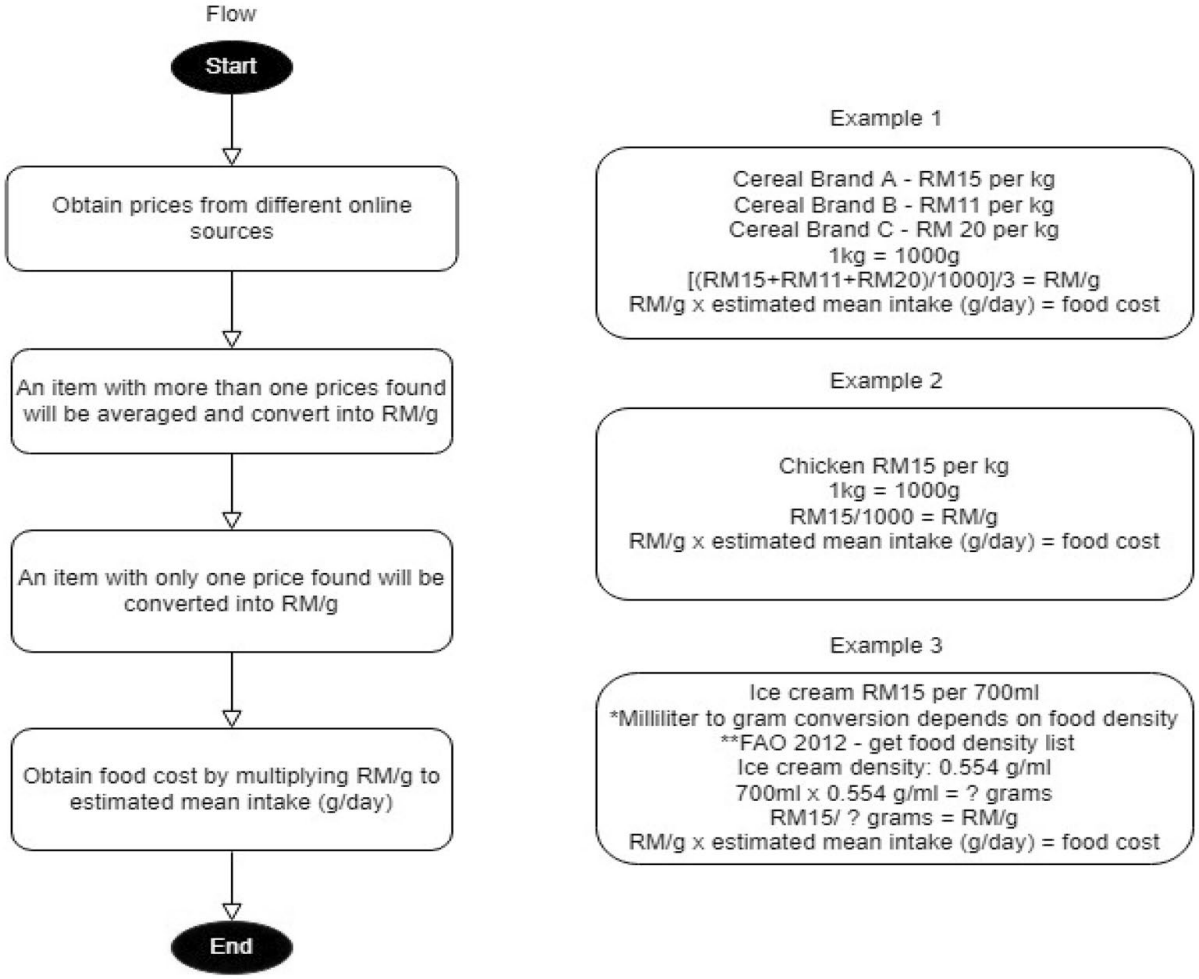
Food cost estimation steps. Source: *Bishop [34]; **FAO [35]

To match with the MANS food intake data, all food cost data are converted from kilograms to grams, whereas for liquidized food items they are converted from milliliters to grams. After that, all cost data are averaged and converted to Ringgit Malaysia (RM) per gram, eventually multiplied by the daily mean intake from MANS to get the total cost estimates. The food cost conversion steps are illustrated in Fig. 2, whereas the estimated upper and lower limits for food cost are listed in Table 3.

**Table 3:** The lower and upper limit of daily per capita food cost

MANS	Cost (RM)	
	Lower limit	Upper limit
2003	9.47	11.99
2014	10.27	13.15

Source: Estimation is based on various sources listed in Appendix 1

## Results and discussions

### Food and nutrient perspectives

The optimized results on per capita and daily basis are shown in Table 4 for MANS 2003 and Table 5 for MANS 2014. Overall, the serving and nutrient goals that were not achieved in the observed dietary intake are met in the optimized diets. The most noticeable result from nutrition perspective is the lack of consumption in calcium nutrient. Malaysian adults are found to have consumed less calcium than the minimum recommendations in both observed years. This is consistent to the lesser quantity intake for milk and vegetables food groups listed in Table 6. Hence, the intake of milk and vegetable products are suggested to increase. Previous research [36] found that income, gender, health condition, and location of residence are highly correlated to low intake of vegetable products. Among them, low income is an expected factor due to consumers are likely to allocate their limited budget to more essential food (such as rice) and non-food items (such as clothing and housing). Also, domestic milk production is slow in its growth in Malaysia and thus of high reliance on imported products. Milk products are not exactly available or affordable by all, which could trigger its low intake [37]. In addition, some studies also found that Malaysians could have high prevalence rate of lactose intolerance [38, 39] and they are advised to consider soybeans and vegetables as alternatives to supplement the intake of calcium.

**Table 4:** Comparison of per capita daily nutrient content between the observed and optimal food intakes by food groups, MANS 2003

Nutrient	Observed per capita daily intake							
	A. Cereals	B. Meat	C. Seafood & eggs	D. Legumes	E. Milk	F. Vegetables	G. Fruits	Total
Energy (kcal)	1176	219	294	356	210	95	212	2562
Protein (g)	31	17	35	22	8	5	4	122
Fat (g)	23	14	14	23	9	1	2	86
Carbohydrate (g)	212	6	6	20	24	17	46	331
Calcium (mg)	132	11	169	93	248	89	61	803
Sodium (mg)	1170	251	371	0	134	11	31	1969
Vitamin A (μg RE)	771	36	176	3	145	361	275	1767
Vitamin C (mg)	9	2	1	4	2	29	151	199
Vitamin B1 (mg)	0.5	0.2	0.2	0.4	0.3	0.1	0.2	1.9

	Optimized per capita daily intake							
	A. Cereals	B. Meat	C. Seafood & eggs	D. Legumes	E. Milk	F. Vegetables	G. Fruits	Total
Energy (kcal)	1068	219	103	81	608	116	136	2332
Protein (g)	29	17	14	7	19	8	2	95
Fat (g)	23	14	4	4	20	1	1	67
Carbohydrate (g)	187	6	4	6	89	20	30	341
Calcium (mg)	129	11	138	53	640	185	44	1200
Sodium (mg)	1165	251	296	0	282	11	21	2027
Vitamin A (μg RE)	771	36	41	3	421	1089	44	2405
Vitamin C (mg)	9	2	1	4	3	43	66	129
Vitamin B1 (mg)	0.5	0.2	0.0	0.1	1.0	0.2	0.1	2.2

Source: This study

**Table 5:** Comparison of per capita daily nutrient content between the observed and optimal food intakes by food groups, MANS 2014

Nutrient	Observed per capita daily intake							
	A. Cereals	B. Meat	C. Seafood & eggs	D. Legumes	E. Milk	F. Vegetables	G. Fruits	Total
Energy (kcal)	1194	148	314	49	119	80	115	2020
Protein (g)	29	11	35	5	5	5	2	91
Fat (g)	21	9	11	2	6	1	1	50
Carbohydrate (g)	223	6	18	4	12	14	25	302
Calcium (mg)	104	7	134	36	151	94	33	560
Sodium (mg)	1,101	183	478	0	45	11	18	1836
Vitamin A (μg RE)	613	18	175	2	72	452	91	1423
Vitamin C (mg)	6	1	1	4	1	31	76	120
Vitamin B1 (mg)	0.3	0.1	0.2	0.1	0.1	0.1	0.1	1.0

	Optimized per capita daily intake							
	A. Cereals	B. Meat	C. Seafood & eggs	D. Legumes	E. Milk	F. Vegetables	G. Fruits	Total
Energy (kcal)	953	203	132	70	550	75	134	2117
Protein (g)	24	14	14	7	21	6	2	88
Fat (g)	16	12	6	2	26	1	1	64
Carbohydrate (g)	180	9	4	6	59	12	30	300
Calcium (mg)	91	10	112	50	715	187	34	1,200
Sodium (mg)	805	277	222	0	165	10	21	1500
Vitamin A (μg RE)	578	22	142	4	327	1,168	96	2336
Vitamin C (mg)	6	1	0	7	5	44	77	140
Vitamin B1 (mg)	0.3	0.1	0.1	0.2	0.5	0.2	0.1	1.5

Source: This study

**Table 6:** Comparison of per capita daily servings and costs between the observed and optimal food intakes by major food groups, MANS 2003 and MANS 2014

Food Groups	Servings/Capita/Day		Cost (RM)/Capita/Day	
	Observed	Optimized	Observed	Optimized
MANS 2003				
A. Cereals	7.05	6.23	4.75	4.54
B. Meat	1.19	1.20	0.84	0.85
C. Seafood and eggs	2.49	1.00	2.37	1.33
D. Legumes	3.09	1.00	1.31	0.55
E. Milk	1.20	2.68	0.77	1.26
F. Vegetables	1.54	3.00	0.48	0.78
G. Fruits	3.05	2.00	1.47	1.18
Total	19.61	17.11	11.99	10.49
MANS 2014				
A. Cereals	7.43	6.00	6.27	4.90
B. Meat	0.76	1.00	0.65	0.83
C. Seafood and eggs	2.47	1.00	2.50	1.29
D. Legumes	0.67	1.00	0.57	0.93
E. Milk	0.66	2.98	0.72	2.93
F. Vegetables	1.64	3.00	1.35	1.12
G. Fruits	1.70	2.00	1.09	1.15
Total	15.33	16.98	13.15	13.15

Source: This study

Next, although Malaysian adults consumed lots of cereal-based foods, their consumptions are still within the recommended range. This is consistent with the findings from MANS reports, in which rice is the top food consumed by the surveyed groups [40, 41]. It is likely attributable to social and cultural norms, whereby rice can be transformed into different types of cuisine and is exceptionally easy to blend into every meal that Malaysian consume, in comparison to the other food items.

On the other hand, seafood and eggs that contribute to major source of protein in the observed diet are encouraged to reduce to an appropriate level as suggested in the optimized diets. The higher intake of seafood and eggs may be due to several reasons, not only the prices but also the conveniences of readily available, easily reachable, and generally acceptable by all ethnicities. Unlike poultry products, pork and beef are prohibited for Muslims and Hindus, in addition to some Chinese that adopt some Buddhism beliefs and do not consume beef. Nevertheless, a meal plan that is inclined to a specific selection may not conform to a balanced diet that is beneficial to health. Thus, a cut in consumption of this food group is suggested.

To be in line with the concept of affordability, we define a target diet that is able to achieve food group servings recommended by MyDG and nutrient content recommended by RNI with reasonable costs. The target diet emerged in which food choices shift is required to commensurate the definition from food and nutrient perspectives. Based on the findings of this study which had referenced the clinical suggestions from guidelines released by the governmental portals, calcium was the nutrient found to be consistently less consumed. For improvement, an increase in the consumption of milk and vegetable products is considered adequate to meet most people’s nutrient need, whereas the consumption of seafood and egg products is suggested to reduce. Nevertheless, it is noted that the consumption of these food items must be based on individual’s health condition and activity levels. Maintaining balanced dietary intake and avoid falling into chronic disease predicaments are the ultimate goals to achieve. Although there could be many factors attached to the chosen diet plan, but from health maintaining perspective, a consistent one is still the income and food cost that are affordable.

### Food cost perspective

The Minimum Wage Order of Malaysia was established in the year 2012. Before the commencement of this law, Poverty Line Income (PLI) was adopted as a measure equivalent to minimum wages. In 2003, the estimated monthly PLI^1^ was RM 610, whereas RM 850 was estimated as the minimum wage in the year 2013^2^, RM 1100 for the year 2019, and RM1 200 for the year 2021.

In row 1 of Table 7, the optimized food cost results indicate that Malaysian adults on average were required to spend RM 10.49 per day on food in the year 2003 and RM 13.15 per day in the year 2013. By linking the optimized food costs to the minimum wage of Malaysia, we obtained total budget on food as RM 314.70 per month, equivalent to 52% of the monthly PLI in the year 2003. However, total food budget for the year 2013 was RM 394.50 per month, equivalent to 46% of the monthly minimum wage. Assuming the optimized intake of the year 2019 and 2021 is the same as the intake of the year 2013, by using inflation rate formula to conduct a forward simulation, we obtain total food budget for the year 2019 and 2021 as RM 468.43 and RM479.04 per month, equivalent to 43% and 40% of the minimum wages. (Table 7, rows 2 and 4).

**Table 7:** Comparison of per capita expenditure on food with monthly minimum income

Description		2003	2013	2019	2021
Optimized food cost per day: (RM/day)	(*a*)	10.49	13.15	15.61	15.97
Optimized food cost per month: (RM/month)	(*b* = a*30)	314.70	394.50	468.43	479.04
Estimated income per month: (RM/month)	(*c*)	610.00	850.00	1100.00	1200.00
Ratio of food expenditure to income: (%)	(*d* = *b*/*c*)	52%	46%	43%	40%

Source: This study

2003 PLI is estimated from Chapter 3 in the Mid-Review of 8th Malaysia Plan [42]. The minimum wages in 2013, 2019, and 2021 are derived from the Minimum Wage Order 2012 [43], 2018 [44], and 2020 [45], effective 1st Jan, 2013 and 2019, and 1st Feb, 2020, respectively

The optimized food cost in 2019 and 2021 is estimated by assuming that the optimal servings in both years are equivalent to the optimized servings in 2013 and food cost inflation rates are equivalent to 18.74% and 21.43%, which are the CPI inflation rates from 2013 to 2019 and 2013 to 2021, respectively

As aforementioned, we defined a target diet as “a diet that is most closely represent a realistic diet as be able to achieve the recommended servings by MyDG and nutrient recommendations by RNI with reasonable cost.” Pertaining to this definition, it is achievable from nutrient perspective with reasonable food choices shift. However, cost on food is rising over time due to inflation. Although the ratio of food expenditure to income is showing a decreasing trend, it is still higher than the figure proposed in the expenditure guideline [16] released by the Employees Provident Fund of Malaysia in 2019. The guideline indicates that a total budget of RM 1870 per month is required for the living of an individual who is single and a public transport user, whereby RM 550 should be allocated to food. The recommended food budget is equivalent to only 29% of the total budget, yet it occupies 50% of the minimum wage, and with our simulated optimized cost, there is still a gap of 14% (43%–29%) even though the optimized cost had been controlled at the observed cost level. Hence, if one’s earning is near or at the minimum wage level, the higher food cost attached to a healthier diet following the nutritional guidelines proposed by the government could raise public concerns on the affordability of healthy diet for low-income group.

In fact, according to the statistics from the Central Bank of Malaysia, the starting salaries for workers with no prior working experience in the non-executive level were all just close to the minimum wage [46]. It was reported that Malaysian’s salaries were found to have misaligned with their productivity levels. With the same level of output produced, Malaysians received lower pay than the workers in the benchmark economies. Researchers [47] also found that Malaysians tended to have prevalence of food insecurity. There was a total of 6 parameters used to measure the prevalence, and 4 of them are believed to be highly correlated to the income level: (1) could not afford to feed children with various food (20.8% prevalence), (2) only rely on cheap food and affordable food to feed children (23.7% prevalence), (3) skipped main meal (15.2% prevalence), and (4) reduced size of meal (21.9% prevalence).

Since COVID-19 pandemic started to outbreak in Malaysia, the gradually revealing income matter could have worsened, the unemployment rate had increased drastically within a short period of time from about 500,000 people to more than 700,000 people unemployed in just 4-month period from January to April 2020 [48]. There were also more than 30,000 businesses closed down as of November 2020 [49]. With the extension of Movement Control Order and lockdown measures enforced by the government to bring down the infectious cases, many Malaysians were suffered from job lost and hence income shrinkage. The tough battle with the disease is going on in 2022 and is expected to continue [50, 51]. While there are proposals [52, 53] to increase the minimum wage level to RM 1,500 per month, the estimated optimized cost per month is equally going to increase as the core inflation rate has set to rise. Hence, wages-relevant laws, such as the Minimum Wage Order, shall position into a more careful assessment that is always up to the par of inflation rate as rising food cost amid the post-pandemic economic recovery could aggravate food insecurity in low-income households.

## Conclusions

We demonstrate that improving dietary quality is possible without increasing the existing cost by using goal programming models. Although acceptability is achievable with food choices shift based on each individual consumption preference, the problem of affordability still remains challenging for low-income households. Income is an indispensable element when we strive to make improvement toward food and nutrition security [54–56]. Research [47] found that education also plays an important role in combating food insecurity. We believed that not only education on nutrition is required, but the most closely linked element to income generation is also education which could aid low-income households to stay out of the poverty cycle. Hence, policies aiming at income growth are likely to benefit the low-income groups in terms of nutrient availability and the quality of their diet [57, 58].

However, we believe our approach could provide valuable information for food and nutrition security program planners in the disadvantaged environments by identifying key problem nutrients and related food costs in the available local diet. There are three limitations of this study that we would like to highlight. First, the data from MANS have the prevalence of underreporting as admitted in the original MANS 2014 report. Hence, the actual nutrient intakes are believed to be higher than reported, which means the food cost in this study could be underestimated. Second, food intake constraints are specified in food groups instead of food subgroups because the referenced data from MyDG is in food groups form. Hence, the constraints are believed to be more stringent. Third, the cost data were estimated based on a mixture of raw and processed food prices without further breaking into condiment granularities consideration. For food, like *roti canai*, the collected data were purely based on available price that could be found online, such as in a forum or travel tips website. Besides that, some prices were mapped by food type similarity, for example, rice and rice porridge were collected from the same source. Hence, the estimated costs are believed to be lower than the actual ones.

Besides the aforementioned limitations, more knowledge is needed on how nutrition information on food products influences consumer choices. In model formulation, consumers’ sensitivity to health information from different food products should also be further investigated. Future research can be designed to assess the impact of alternative promotion strategies to achieve more balanced diets for a more prosperous population in Malaysia.

## Data Availability

The datasets used and/or analyzed during the current study are available from the corresponding author on request.

## Appendix 2. Mapping of food groups and subgroups in MANS 2003 and MANS 2014

**Table:**

Food groups	Food subgroup items		
	MANS 2003	MANS 2014	English translation that available in MANS 2014
1. Cereals	Bijirin	Bijirin sarapan pagi	Cereals
	Biskut	Biskut berperisa/berkrim/berinti	Flavored/ cream/ filled cookies
		Biskut tawar/krim kraker	Cream crackers
	Mihun/kueh teow/laksa/laksam	Mihun/Kueh teow/laksa/laksam/loh shi fun	Rice vermicelli/Rice noodle/Loh Shi Fun
	Loh Shi Fun		
	Nasi	Nasi beras perang	Brown rice
		Nasi berperisa	Flavored rice
		Nasi putih	White rice
	Bijirin tersedia perlu dibancuh	Bijirin tersedia perlu dibancuh	Instant cereal
	Bubur Nasi	Bubur nasi	Rice porridge
	Capati	Capati	Chapati
	Mee kuning/mee siput/mee segera	Mee kuning/mee siput/mee segera	Wheat Noodles
	Pasta	Pasta	Pasta
	Pizza	Pizza	Pizza
	Pulut	Pulut	Glutinous rice
	Roti	Roti	Bread
	Roti Bun	Roti bun	Bun
	Roti canai	Roti canai	Roti canai
	Tosai	Tosai	Tosai
2. Meats	Bebola ayam/ketam/udang	Bebola ayam	Chicken ball
	Ayam	Ayam	Chicken
	Babi	Babi	Pork
	Bacon	–	Bacon
	Itik	Itik	Duck
	Kambing	Kambing	Mutton
	Lembu/Kerbau	Lembu/kerbau	Meat
	Luncheon meat	Luncheon meat	Luncheon meat
	Nugget	Nugget	Nugget
	Sosej/hotdog/frankfurthe	Sosej/hotdog/frankfurter	Sosej/hotdog/frankfurter
3. Seafood & Eggs	Bebola ikan/kek ikan	Bebola/kek ikan/udang/sotong/ketam	fish/ prawn/ squid/ crab ball or cake
	Ikan air tawar	Ikan air tawar	Freshwater fish
	Ikan bilis	Ikan bilis	Anchovy
	Ikan dalam tin	Ikan dalam tin	Canned fish
	Ikan kering	Ikan kering	Dried fish
	Ikan laut	Ikan laut	Marine fish
	Kekerang	Kekerang	Shellfish
	Keropok lekor	Keropok lekor	Keropok lekor
	Ketam	Ketam	Crab
	Snek/keropok/kerepek	Snek/kerepek	Snacks/Crackers
	Sotong basah	Sotong basah	Squid
	Udang basah	Udang basah	Prawn
	Telur ayam	Telur ayam	Chicken eggs
	Telur masin	Telur masin	Salted eggs
	Telur puyuh	–	Quail eggs
4. Legumes	Sayuran kacang	Sayuran kekacang lain	Other type of legumes
	Kacang tanah	Kacang tanah	Groundnuts
	Kekacang	Kekacang	Legumes
	Tauhu	Tauhu	Tofu
	Tempe	Tempe	Fermented soy beans
5. Milk	Aiskrim susu	Aiskrim (susu)	Ice cream
	Keju	Keju	Cheese
	Krim keju	–	Cream cheese
	Susu segar/UHT	Susu segar	Fresh milk
	Susu pekat manis	Susu pekat manis	Condensed milk
	Susu sejat/cair	Susu sejat/cair	Evaporated milk
	Susu tepung	Susu tepung	Powdered milk
6. Vegetables	Cendawan basah/kering	Cendawan basah	Mushrooms
		Cendawan kering	Dried mushrooms
	Jagung	Jagung	Maize (corn)
	Sayuran asin/kering	Sayuran asin/kering	Salted or dried vegetables
	Sayuran berdaun hijau	Sayuran berdaun hijau	Leafy green vegetables
	Sayuran berubi	Sayuran berubi	Tubers
	Sayuran kobis	Sayuran kobis	Cabbages
	Taugeh	Taugeh	Bean sprout
	Ulam-ulam	Ulam-ulaman	Local fresh salads
7. Fruits	Longan segar	Mata kucing segar	Longan
	Anggur	Anggur	Grape
	Belimbing	Belimbing	Starfruit
	Betik	Betik	Papaya
	Buahan dalam tin	–	Canned fruits
	Buahan kering	Buahan kering	Dried fruits
	Durian	Durian	Durian
	Epal	Epal	Apple
	Jambu Batu	Jambu batu	Guava
	Mangga	Mangga	Mango
	Nenas	Nenas	Pineapple
	Oren/Mandarin	Oren/Mandarin	Orange
	Pir/Lai	Pir/Lai	Pear
	Pisang	Pisang	Banana
	Tembikai	Tembikai	Watermelon
	Tembikai susu	Tembikai susu	Honey dew

“– ” denotes food subgroup was excluded from modeling due to low intake, that is, intake value was zeroed after rounded off to two decimal places

## Abbreviations

FAO: Food and Agriculture Organization
UNICEF: United Nations Children's Fund
IFPRI: International Food Policy Research Institute
NPAN: National Plan of Action for Nutrition
EPF: Employees Provident Fund
MANS: Malaysian Adult Nutrition Survey
MyDG: Malaysian Dietary Guidelines
MyFCD: Malaysian Food Composition Database
RNI: Recommended Nutrient Intakes
CPI: Consumer Price Index
DOSM: Department of Statistics Malaysia
RM: Ringgit Malaysia
PLI: Poverty Line Income
Covid-19: Coronavirus disease 2019

## Footnotes

1 Only Poverty Line Income for the year 2002 was found. Therefore, it was used to represent 2003 instead.

2 Selected the year 2013 instead of 2014 is to comply with one of the objectives mentioned in MANS 2014, which was to assess the trend of Malaysian dietary intake between the year 2003 and 2013 (10-year interval). MANS 2014 was a follow-up to the MANS 2003 and the data collection for MANS 2014 was at the beginning of the year, whereas MANS 2003 was a yearlong.
